# Inhibition of the PI3K-AKT-mTOR pathway suppresses the adipocyte-mediated proliferation and migration of breast cancer cells

**DOI:** 10.7150/jca.37975

**Published:** 2020-02-10

**Authors:** Jae-Yeo Park, Shi-Eun Kang, Kwang Seok Ahn, Jae-Young Um, Woong Mo Yang, Miyong Yun, Seok-Geun Lee

**Affiliations:** 1 Department of Science in Korean Medicine and Comorbidity Research Institute, Kyung Hee University, Seoul, Republic of Korea; 2 KHU-KIST department of Converging Science and Technology, Kyung Hee University, Seoul, Republic of Korea; 3 Bionanocomposite Research Center, Kyung Hee University, Seoul, Republic of Korea; 4 Department of Bioindustry and Bioresource Engineering, College of Life Sciences, Sejong University, Seoul, Republic of Korea; 5 Sejong Arctic Research Center, Sejong University, Seoul, Republic of Korea

## Abstract

**Objective**: Although it is well known that adipocyte significantly affects breast cancer progression, its mechanism has not been fully understood. Here, we analyzed the effect of adipocytes on breast cancer progression including cell proliferation and migration.

**Materials and Methods**: We treated the conditioned media obtained from mouse 3T3-L1-derived or human adipose tissue-derived mesenchymal stem cells (hAMSC)-derived adipocytes to breast cancer cells, MCF-7 and MDA-MB-231. And then, cells viability and proliferation were analyzed using MTT assays and colony forming assays, respectively. Also mRNA expression of inflammatory cytokines and proteins expression in main signal pathway were analyzed by RT-qPCR and immunoblotting, respectively.

**Results**: Adipocyte-derived conditioned media increased the proliferation and migration of MCF-7 and MDA-MB-231 cells while little effects in a human normal immortalized mammary epithelial cell line MCF10A. In addition, adipocyte-derived conditioned media induced phosphorylation of AKT and mTOR and upregulated the expression of target genes of the PI3K-AKT-mTOR pathway including IL6, IL1β, IL1α and TNFα in breast cancer cells. Furthermore, BEZ235 a dual inhibitor of PI3K and mTOR significantly decreased the adipocyte-mediated the proliferation and migration of breast cancer cells.

**Conclusion**: Adipocyte-derived conditioned media enhance the proliferation and migration of breast cancer cells through the PI3K-AKT-mTOR pathway, supporting the importance of heterotypic interactions between breast cancer cells and adipocytes in the tumor microenvironment.

## Introduction

Breast cancer has been the most commonly diagnosed cancer and the leading cause of cancer death in women over the world 1. Recent Investigation of the estimated numbers of new cancer cases and deaths in the United States in 2019 noticed that breast cancer is again expected to be the most common cancer accounting for 30% of all new cancer diagnoses and the second leading cause of cancer-related death in women 2.

Fibroblasts, endothelial cells and immune cells in the tumor microenvironment have been paid attention because their heterotypic interactions with cancer cells or themselves play major roles in cancer growth and progression 3, 4. However, adipocytes, a major component of the normal breast and the tumor microenvironment in breast cancer, have been increasingly recognized as another dominant player for breast cancer progression. Recent studies using animal models, *in vitro* co-culture systems and adipocyte-derived conditioned media have suggested that cancer-associated adipocytes stimulate the proliferation, invasion, migration, and metastasis of breast cancer as well as its resistance to various therapeutic treatments including chemotherapy and radiotherapy 5-7. Adipocyte-derived soluble factors called adipokines including adiponectin, leptin, IL-6, IL1β, and TNFα have known to be involved in the breast cancer progression through their receptors 6, 8. AMPK is noticed to regulate the expression of IL-6 and IL-8 in adipocytes, suggesting that targeting AMPK in adipocytes could be a novel way to modulate obesity-related adipokine production associated with insulin resistance and breast cancer progression 9. In addition, adipocytes, as well as obesity in breast cancer patients, are closely related with the increased resistance to chemotherapy including trastuzumab and docetaxel leading to the challenges for the clinical management of the tumor 10. Recent studies have focused on what signaling molecules adipokines regulate in the pathogenesis of breast cancer. Adipocytes-derived leptin and IL-6 have known to activate Jak/STATs and PI3K/AKT pathways and to increase the expression of Lysyl Hydroxylase-2 in breast cancer cells leading to tumor progression including epithelial-mesenchymal transition and metastasis 11, 12. However, more detailed molecular mechanisms of the adipocyte-induced tumor progression still need to be uncovered yet in breast cancer.

The PI3K-AKT-mTOR pathway is one of the most frequently dysregulated pathways in the pathogenesis of breast cancer associated with crucial cellular functions including survival, proliferation, invasion, and metabolism as well as with poor clinical outcome of the patients 13, 14. The PI3K-AKT-mTOR pathway is generally initiated by the interaction of PI3K with either G-protein coupled receptors (GPCRs) 15 or receptor tyrosine kinases (RTKs) such as insulin-like growth factor 1 receptor (IGF-1R) 16 and ErbB family receptors 17. Recent findings have suggested that the PI3K-AKT is involved in the adipokine-induced tumorigenesis in breast cancer 12, 17. In the present study, we investigated the importance of the PI3K-AKT-mTOR signaling pathway for the adipocyte-induced breast cancer cell proliferation and migration using the adipocyte-derived conditioned media.

## Materials and Methods

**Reagents and cell culture.** All processes for cell culture experiments were followed by standard Biosafety Level 2 (BSL‑2) guidelines. All cell lines used in this study were tested whether there is any mycoplasma using mycoplasma detection kits (JCBIO, Songpa-gu, Seoul, South Korea). Human breast cancer MCF-7 and MDA-MB-231 cell lines and a human normal mammary epithelial MCF10A cell line were previously described 18. All cells were cultured in a humidified incubator with 5% CO_2_ at 37˚C and the viability of cultured cells was monitored using a LUNA-FL Automated cell counter (Logos Biosystems, Inc., Anyang, Republic of Korea). BEZ235 was purchased from Selleckchem (Houston, TX, USA). Insulin and dexamethasone were purchased from Sigma Chemical Co (St. Louis, MO, USA). All other chemicals and reagents without notice were purchased from Sigma Chemical Co (St. Louis, MO, USA).

**Conditioned media derived from 3T3-L1- and hAMSC-differentiated adipocytes.** 3T3-L1 cells were kindly provided by Dr. Hyang-Kyu Lee (College of Nursing, Yonsei University). 3T3-L1 preadipocytes were maintained in DMEM containing 10% calf serum at 37℃ under 5% CO_2_ atmosphere. After reaching the confluence, the differentiation of 3T3-L1 preadipocytes to adipocytes was induced by treatment with insulin (1 μg/ml), dexamethasone (1 μM), and 3-isobutyl-1-methylxanthine (500 μM) in DMEM containing 10% FBS (Thermo Fisher Scientific, Gibco FBS 26140-079, Waltham, MA USA) for 2 days. The induction medium was replaced with DMEM containing 10% FBS and insulin (1 μg/ml) and the cells were cultured for two more days. Then the cells were washed with PBS and replaced with fresh DMEM containing 10% FBS every other day. Conditioned media (CM) from mouse 3T3-L1-derived adipocytes (mCM) were collected 8 days after differentiation. Human adipose tissue-derived mesenchymal stem cells (hAMSC) and the preparation of CM from hAMSC-derived adipocytes (hCM) were previously described 19.

**Cell viability assays.** Cells were plated in 96-well plate (3-5 × 10^3^ cells/well) one day before treatment. Following treatment with conditioned media for 24, 48 and 72 hours, cell viability was determined by 3-(4,5dimethylthiazol-2-yl)-2,5-diphenyltetrazolium bromide (MTT) assays as previously described 18. Absorbance was measured at 490 mm in a microplate reader (Molecular Devices, Mountain View, CA, USA).

**Colony formation assays.** Cells were seeded into 24-well plates (100 cells/well) in 1 ml of medium containing 10% FBS, and one day later were treated with conditioned media (mCM or hCM) for 2 weeks. The cells were stained for 30 min with a solution containing 0.5% crystal violet and 25% methanol, followed by three-time rinses with tap water to remove excess dye.

**Wound healing assays.** Cells were seeded into six-well plates and incubated for 24 hours until reaching ~90% confluency. 200-μl pipette tips were used to make scratches in the center of each well. Several regions were marked and photographed (×20 magnification) at 0 and 18 hours after incubating the scratched cells with mCM or hCM.

**Quantitative real-time PCR.** Total RNA from the cells treated with mCM or hCM for 24 hours was isolated using TRI reagent solution (Ambion, Waltham, MA, USA). The isolated total RNA (1 μg) was reverse transcribed to cDNA using a cDNA Synthesis Kit (DR01611, MGMED, Inc., Seoul, Republic of Korea) according to the manufacturer's instruction. Amplification of each cDNA was analyzed using SYBR Premix Ex Taq (Takara Korea Biomedical Inc., Seoul, Republic of Korea) on the StepOnePlus Real-Time PCR system (Thermo Fisher Scientific Ltd., Waltham, MA, USA). Specific primers used for the amplification of each gene are as following: TNFα (forward: 5'-GACAAGCCTGTAGCCCATGTTGTA-3' and reverse: 5'-CAGCCTTGGCCCTTGAAGA-3'), IL-6 (forward: 5'-GTGGAGATTGTTGCCATCAACG-3' and reverse: 5'-CAGTGGATGCAGGGATGATGTTCTG-3'), MCP1 (forward: 5'-AGCAGCAAGTGTCCCAAAGA-3' and reverse: 5'-GGTGGTCCATGGAATCCTGA-3'), IL1α (forward: 5'-CGCCAATGACTCAGAGGAAGA-3' and reverse: 5'-AGGGCGTCATTCAGGATGAA-3'), IL1β (forward: 5'-CTTTGAAGCTGATGGCCCTAAA-3' and reverse: 5'-AGTGGTGGTCGGAGATTCGTA-3'), IGF1 (forward: 5'-TCGCATCTCTTCTATCTGGCCCTGT-3' and reverse: 5'-GCAGTACATCTCCAGCCTCCTCAGA-3'), VEGF (forward: 5'-AAGCCAGCACATAGGAGAGATGA-3' and reverse: 5'-TCTTTCTTTGGTCTGCATTCACA-3') and GAPDH (forward: 5'-GCACCGTCAAGGCTGAGAAC-3' and reverse: 5'-TGGTGAACACGCCAGTGGA-3'). GAPDH was used as an internal control, and all reactions were always performed in duplicate or triplicate at least three times.

**Western blot analysis.** Whole cell lysates were prepared, and western blot analysis was performed as previously described 18. Primary antibodies against STAT3, p-STAT3, mTOR, p-mTOR, AKT, p-AKT, AMPK, p-AMPK were purchased from Cell Signaling Technology (Danvers, MA, USA), and β-actin from Sigma-Aldrich (Oakville, Canada). Secondary antibodies HRP-conjugated anti-mouse IgG and anti-rabbit IgG (1:5000-1:10000; Santa Cruz Biotechnology, Dallas, USA) were used for western blotting. The blots were developed using EZ-Western Lumi Pico Kit (DoGEN, Seoul, Republic of Korea).

**Statistical analysis.** Data are presented as mean ± standard deviation (SD). Differences between the means of each group were analyzed using Excel's *t*-test and *P* values of <0.05 were considered significant. The statistical software package Excel (Microsoft, WA, USA) was used for the analysis.

## Results

**Figure 1:**
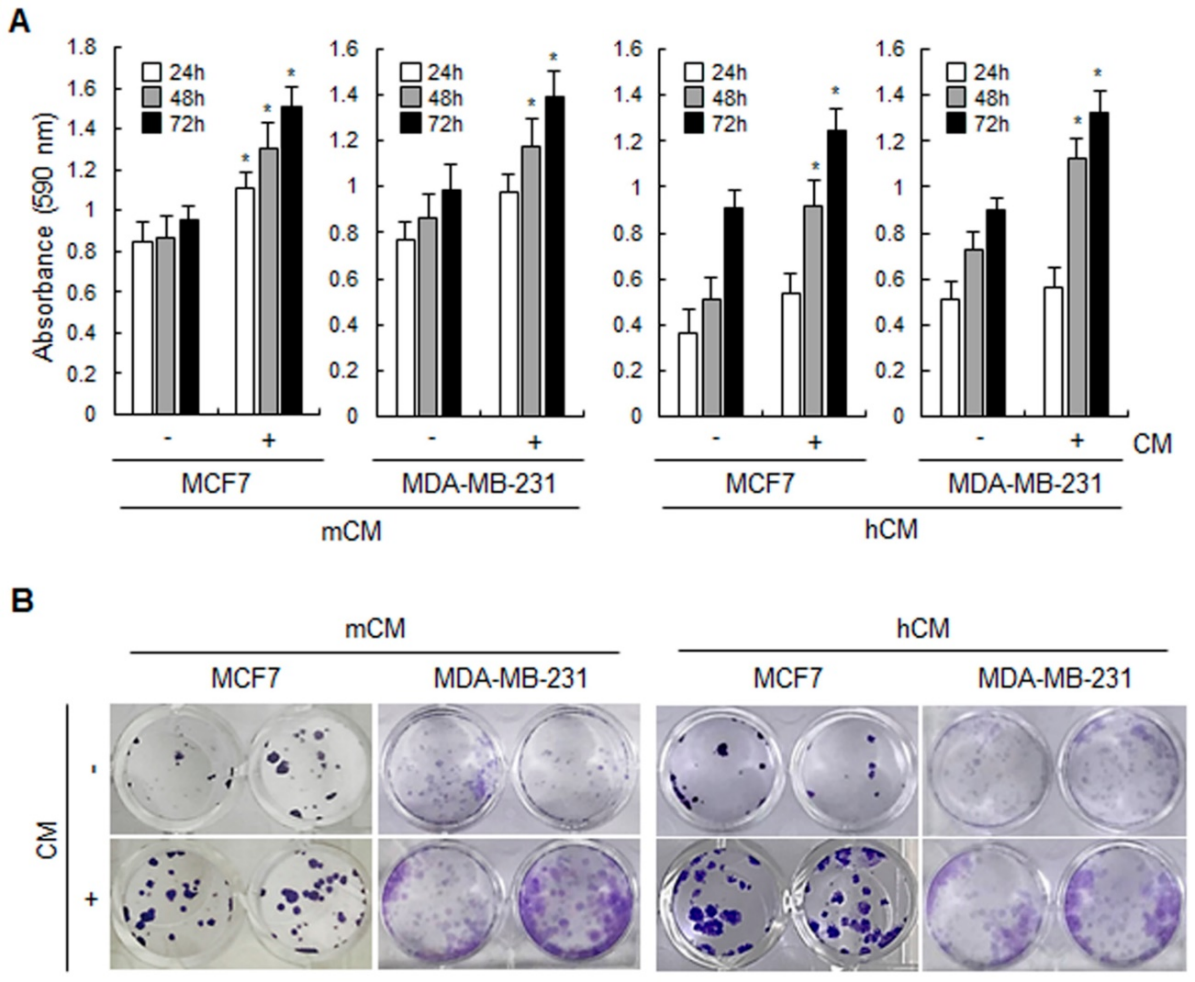
** Effects of mCM and hCM on the proliferation of breast cancer cells.** (A) MCF-7 and MDA-MB-231 cells were treated with mCM or hCM for 24, 48 and 72 hours. Cell viability was then analyzed using MTT assays. Data are presented as the mean ± SD (*P < 0.05 versus mock-treated control). (B) Cell proliferation of MCF-7 and MDA-MB-231 was evaluated by colony forming assays. Cells were seeded into 24-well plates and then treated with mCM or hCM for 2 weeks. Colonies were visualized by crystal violet staining. - and + means without and with CM, respectively.

**Adipocyte-derived conditioned media increase the proliferation of breast cancer cells.** To investigate the effect of adipocyte-derived CM on breast cancer cells, mCM and hCM were prepared from adipocytes differentiated from mouse 3T3-L1 and hAMSC, respectively. As shown in Figure 1A, both mCM and hCM increased the proliferation of breast cancer MCF-7 and MDA-MB-231 cells in MTT assays. However, mCM did slightly enhance the proliferation of normal human mammary epithelial MCF10A cells but no statistical significance (Supplementary Fig. 1A). Similarly, both CMs induced more colonies in MCF-7 and MDA-MB-231 cells, but little effect of mCM on MCF10A cells in colony formation assays (Fig.1B and Supplementary Fig. 1B). These results clearly informed that adipocyte-derived CM positively regulates the proliferation of breast cancer cells.

**Figure 2:**
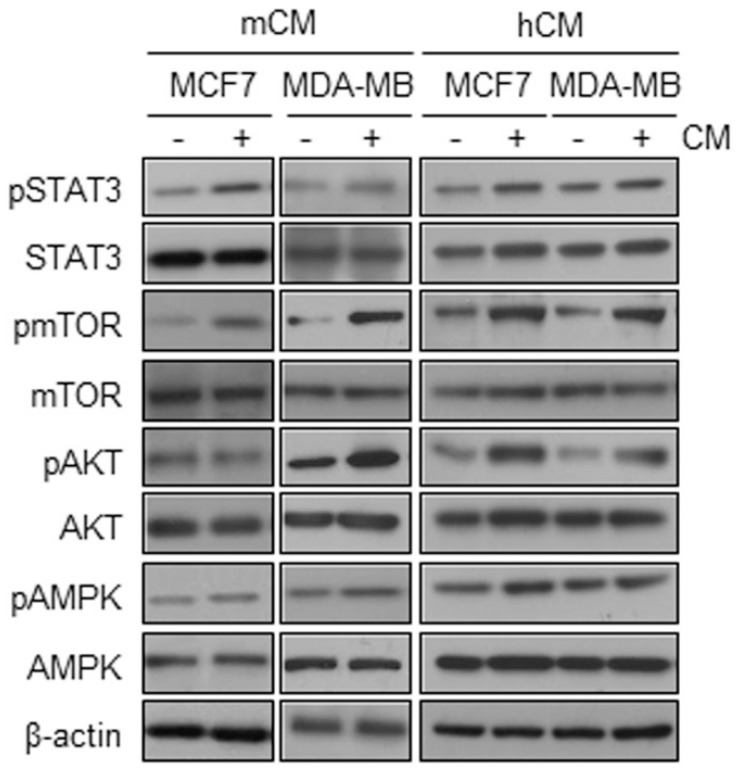
** Effects of mCM and hCM on the activation of AMPK, AKT, mTOR, and STAT3 in breast cancer cells.** MCF-7 and MDA-MB-231 cells were treated with mCM or hCM for 24 hours. Whole cell lysates were prepared and subjected to Western blotting with the indicated antibodies. β-actin was used as an internal control.

**Adipocyte-derived conditioned media dominantly activate the PI3K-AKT-mTOR pathway.** To determine the mechanism by which CMs increases the proliferation of breast cancer cells, we evaluated the activation of upstream signaling molecules related to inflammation- and metabolism-induced proliferation of breast cancer cells. In MCF10A cells, mCM upregulated the phosphorylation levels of only AKT, but not others STAT3, mTOR and AMPK (Supplementary Fig. 1C). However, both mCM and hCM significantly increased the phosphorylation levels of AKT and mTOR in MCF-7 and MDA-MB-231 cells while little effects on the phosphorylation of STAT3 and AMPK (Fig.2). These results suggested that adipocyte-derived CM preferentially induces the PI3K-AKT-mTOR signaling pathway in breast cancer cells.

**Figure 3:**
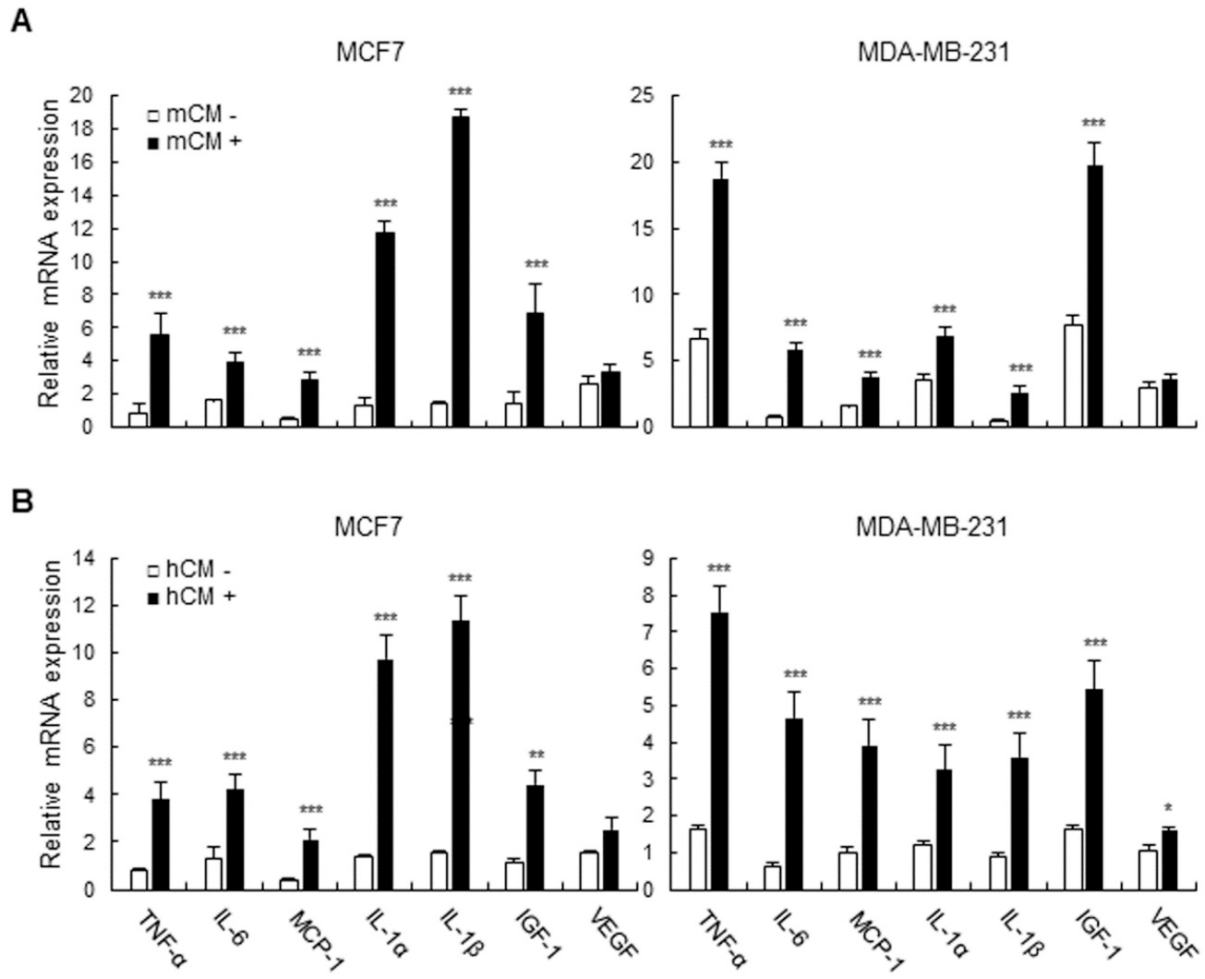
** Effects of mCM and hCM on the expression of cytokines and growth factors in breast cancer cells.** MCF-7 and MDA-MB-231 cells were treated with mCM (A) or hCM (B) for 24 hours. Total RNA was isolated from the treated cells, and mRNA expression of TNF-α, IL-6, MCP-1, IL-1α, IL-1β, IGF-1 and VEGF was analyzed by RT-qPCR. GAPDH was used as an internal control to normalize the expression level of each gene. Data are presented as the mean ± SD (*P < 0.05, ** *P* < 0.01, and *** *P* < 0.001 versus mock-treated control).

**Adipocyte-derived conditioned media induce the expression of inflammatory cytokines and growth factors in breast cancer cells**. In the tumor microenvironment, various secretory molecules including cytokines and growth factors play critical roles for communication between tumor and the surrounding cells such as immune cells, fibroblasts, and adipocytes. Especially, tumor-secreted cytokines and growth factors extensively influence the surrounding cells and itself in the context of immune suppression and autologous activating signals for growth, survival, and invasion 20, 21. We then examined the expression of inflammatory cytokines and growth factors which are target genes of the PI3K-AKT-mTOR pathway in mCM- or hCM-treated breast cancer cells. As shown in Figure 3A and B, mCM and hCM greatly increased the expression of IL-6, IL-1β, IL-1α, TNF-α, IGF-1, and MCP-1 compared with control at the mRNA levels in MCF-7 and MDA-MB-231 cells, while the little effect on the VEGF expression in mCM treated-breast cancer cells. These results indicated that adipocyte-derived CM increases the expression of inflammatory cytokines and growth factors regulated by the PI3K-AKT-mTOR pathway in breast cancer cells, suggesting that the tumor-derived molecules may modulate the tumor microenvironment as well as tumor itself for aggravating the disease.

**Figure 4:**
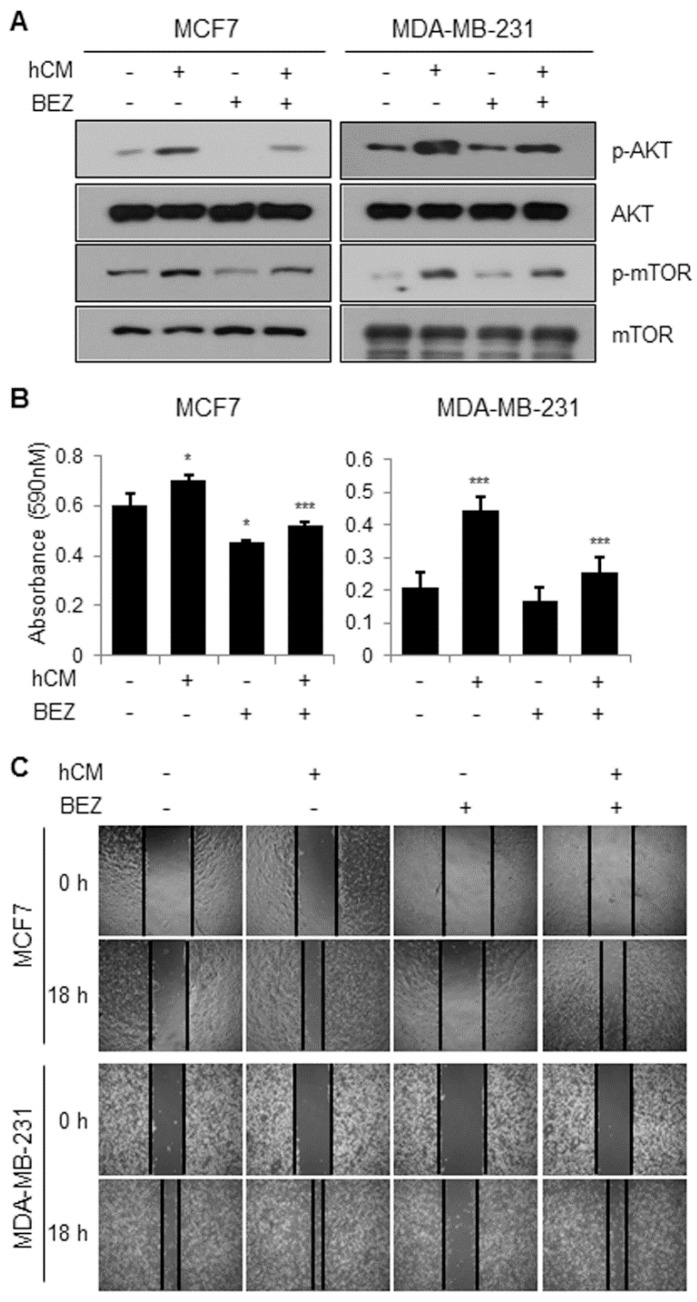
** Effects of a dual PI3K and mTOR inhibitor BEZ235 on the adipocyte-mediated breast cancer cell proliferation and migration.** (A) MCF-7 and MDA-MB-231 cells were treated with hCM in the presence or absence of BEZ235 for 24 hours. Whole cell lysates were prepared and subjected to Western blotting with the indicated antibodies. (B) MCF-7 and MDA-MB-231 cells were treated with hCM in the presence or absence of BEZ235 for 72 hours. Cell viability was then analyzed using MTT assays. Data are presented as the mean ± SD (*P < 0.05, ** P < 0.01, and *** P < 0.001 versus mock-treated control. hCM + BEZ was compared with hCM only). (C) MCF-7 and MDA-MB-231 cells were seeded into 6-well plates and incubated until reaching ~90% confluency. The cells were then scratched in the center of each well, and treated with hCM in the presence or absence of BEZ235 for 18 hours. Several regions were marked and photographed (×20 magnification) at 0 and 18 hours after the indicated treatment.

**BEZ235, a dual inhibitor of PI3K and mTOR inhibits the adipocyte-derived CM-mediated breast cancer progression.** We then confirmed whether the PI3K-AKT-mTOR pathway is involved in the adipocyte-derived CM-induced cell proliferation. As shown in Figure 4A, BEZ235 clearly reduced the hCM-induced phosphorylation of AKT and mTOR in MCF-7 and MDA-MB-231 cells. In addition, BEZ235 decreased the hCM-induced proliferation of breast cancer cells (Fig.4B). Furthermore, we found that hCM enhanced the migration of MCF-7 and MDA-MB-231 cells and BEZ235 inhibited the hCM-induced migration of the breast cancer cells (Fig.4C). These results noticed that the PI3K-AKT-mTOR pathway plays a critical role in the adipocyte-derived CM-mediated proliferation and migration of breast cancer cells.

## Discussion

Estrogen receptor (ER), progesterone receptor (PR) and human epidermal growth factor 2 (HER2/neu) are essential biomarkers in breast cancer. Standard pathological features seem adequate to define clinically useful groups depending on the status of the three biomarkers such as triple-negative, hormone receptor-negative and HER2-positive and hormone receptor-positive\and HER2-positive tumors for which treatment recommendations are seldom controversial 22. Although improvement of therapeutic strategies for breast cancer over the last decades has led to improved survival rates of breast cancer patients, breast cancer is still the leading cause of cancer death due to a variety of hurdles including drug resistance, recurrence and severe side effects 1.

Recent studies have noticed that the adipocyte-derived tumor microenvironment plays crucial roles in tumor progression of various types of human cancer including breast cancer 5, 7, 9, 23. We in the present study also notice that adipocyte-derived CM stimulate the proliferation and motility of breast cancer cells. These results suggest that adipocytes in the tumor microenvironment play an important role in breast cancer progression through the heterotypic interactions between adipocytes and tumor cells. In addition, we present that the PI3K-AKT-mTOR pathway is crucial for CM-mediated the proliferation and migration of breast cancer cells. The PI3K-AKT-mTOR pathway is a complicated intracellular signaling pathway, which responds to nutrients, hormones and growth factors and plays various significant roles in tumor progression including proliferation, motility, survival, angiogenesis and drug resistance 12-14, 22. Because of the importance of the PI3K-AKT-mTOR pathway in the pathogenesis of breast cancer, many targeted inhibitors are currently in clinical trials. Inhibitors of PI3K/AKT/mTOR are classified into four categories: PI3K inhibitors, AKT inhibitors, mTOR inhibitors, and dual PI3K/mTOR inhibitors 24. The most popular inhibitor for mTOR is rapamycin that directly binds to its kinase active domain 25. The classical inhibitors of PI3K are wortmannin and LY294002 that decreased cellular proliferation and induced apoptosis in both *in vitro* and animal models 26. Development of dual inhibitors such as targeting PI3K and mTOR has been intensively invested 27. Accordingly, BEZ235, a PI3K and mTOR dual inhibitor has been investigated as a possible treatment in breast cancer by inducing cell cycle arrest and apoptosis 28-31. However, the pathway and the inhibitor BEZ235 has not been analyzed in the context of heterotypic interactions between breast cancer cells and adipocytes, which are main cellular components especially in obese conditions. A recent review noticed that the efficacy of cancer treatments is significantly lower in obese breast cancer patients 32. Our results in the present study suggest that the PI3K-AKT-mTOR pathway is crucial in the adipocyte-mediated breast cancer progression and that dual inhibitors targeting PI3K and mTOR such as BEZ235 could be a good option to inhibit the obesity-breast cancer link. Further studies with clinical samples of breast cancer patients under between normal and obesity conditions are warranted to provide clinical relevance of our findings and then to pave the way for developing novel therapeutic interventions for the disease under various conditions.

## Conclusions

Taken together, we support that adipocytes in the tumor microenvironment induce breast cancer progression through activating the PI3K-AKT-mTOR signaling pathway, suggesting inhibition of dual target such as PI3K and mTOR as a potentially good option for developing a novel targeted therapy for breast cancer.

## Supplementary Material

Supplementary figure.

## Abbreviations

CBR1: carbonyl reductase 1
HNSCC: head and neck squamous cell carcinoma
LNM: lymph node metastasis
IHC: immunohistochemistry
EMT: epithelial-mesenchymal transition
ROS: reactive oxygen species
ONE: 4-oxonon-2-enal
HNE: 4-hydroxynon-2-enal
PALS1: protein associated with lin seven 1
α-SMA: α-smooth muscle actin
FAP: fibroblast activation protein
Twist: twist family BHLH transcription factor 1
ZEB1: zinc finger e-box binding homeobox 1
NF-kB: nuclear factor kappa B
MMP-7: matrix metalloproteinase 7
ECM: extracellular matrix
VEGF: vascular endothelial growth factor
STAT3: signal transducer and activator of transcription 3
SOD: superoxide dismutase
GPX: glutathione peroxidase
