# Supplementary material for: Inhibition of the PI3K-AKT-mTOR pathway suppresses the adipocyte-mediated proliferation and migration of breast cancer cells
Source: J Cancer. 2020 Feb 10;11(9):2552–9. doi: 10.7150/jca.37975 (PMC7065999; doi:10.7150/jca.37975)
Supplement: Supplementary file 1 — Supplementary figure. [file jcav11p2552s1.pdf]

Supporting information

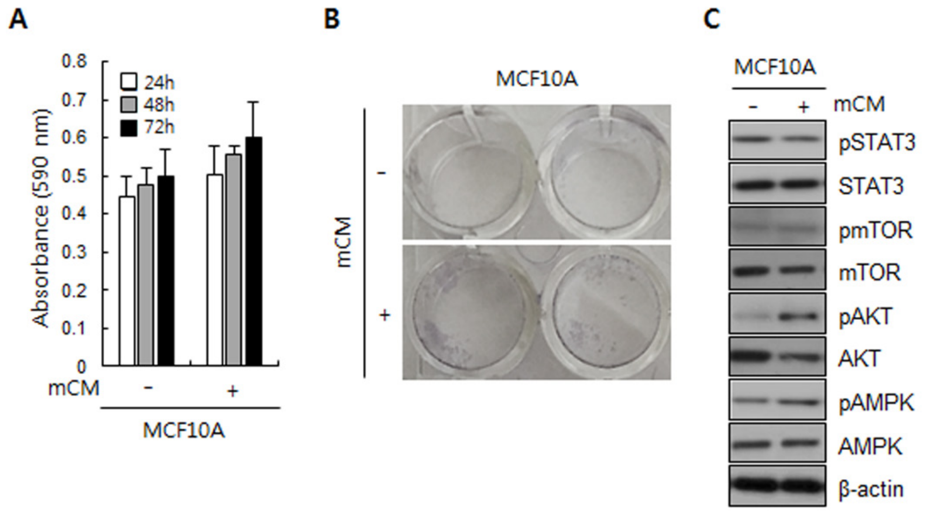

Supplementary Figure 1. Effects of mCM on the cell proliferation and the activation of AMPK, AKT, mTOR and STAT3 in MCF10A cells. (A) MCF10A cells were treated with mCM for 24, 48 and 72 hours. Cell viability was then analyzed using MTT assays. Data are presented as the mean ± SD. (B) Cell proliferation of MCF10A was evaluated by colony forming assays. Cells were seeded into 24-well plates, and then treated with mCM for 2 weeks. Colonies were visualized by crystal violet staining. (C) MCF10A cells were treated with mCM for 24 hours. Whole cell lysates were prepared and subjected to Western blotting with the indicated antibodies. β-actin was used as an internal control.
